# Dance Movement Therapy for Clients With a Personality Disorder: A Systematic Review and Thematic Synthesis

**DOI:** 10.3389/fpsyg.2021.581578

**Published:** 2021-03-18

**Authors:** S. T. Kleinlooh, R. A. Samaritter, R. M. van Rijn, G. Kuipers, J. H Stubbe

**Affiliations:** ^1^Department of Arts Therapies, Codarts University of the Arts, Rotterdam, Netherlands; ^2^KenVaK Research Centre for the Arts Therapies and Psychomotricity, Heerlen, Netherlands; ^3^Professorship Performing Arts Medicine, Codarts University of the Arts, Rotterdam, Netherlands; ^4^PErforming artist and Athlete Research Lab (PEARL), Rotterdam, Netherlands; ^5^Antes^PG^, Parnassia Group, The Hague, Netherlands; ^6^Department of General Practice, Erasmus MC University Medical Center, Rotterdam, Netherlands

**Keywords:** dance therapy, dance movement therapy, arts therapies, creative arts therapies, personality disorder

## Abstract

**Background:** People with a personality disorder (PD) suffer from enduring inflexible patterns in cognitions and emotions, leading to significant subjective distress, affecting both self and interpersonal functioning. In clinical practice, Dance Movement Therapy (DMT) is provided to clients with a PD, and although research continuously confirms the value of DMT for many populations, to date, there is very limited information available on DMT and PD. For this study, a systematic literature review on DMT and PD was conducted to identify the content of the described DMT interventions and the main treatment themes to focus upon in DMT for PD.

**Methods:** A systematic search was conducted across the following databases: EMBASE, MEDLINE, PubMed, WEB OF SCIENCE, PsycINFO/OVID, and SCOPUS following the PRISMA guidelines. The Critical Appraisal Skills Programme for qualitative studies was used to rank the quality of the articles. The Oxford Center for Evidence-based Medicine standards were applied to determine the hierarchical level of best evidence. Quantitative content analysis was used to identify the intervention components: intended therapeutic goals, therapeutic activities leading to these goals, and suggested therapeutic effects following from these activities. A thematic synthesis approach was applied to analyze and formulate overarching themes.

**Results:** Among 421 extracted articles, four expert opinions met the inclusion criteria. Six overarching themes were found for DMT interventions for PD: self-regulation, interpersonal relationships, integration of self, processing experiences, cognition, and expression and symbolization in movement/dance. No systematic descriptions of DMT interventions for PD were identified. A full series of intervention components could be synthesized for the themes of self-regulation, interpersonal relationships, and cognition. The use of body-oriented approaches and cognitive strategies was in favor of dance-informed approaches.

**Conclusions:** Dance movement therapists working with PD clients focus in their interventions on body-related experiences, non-verbal interpersonal relationships, and to a lesser extent, cognitive functioning. A methodological line for all intervention components was synthesized for the themes of self-regulation, interpersonal relationships, and cognition, of importance for developing systematic intervention descriptions. Future research could focus on practitioners' expertise in applying DMT interventions for PD to develop systematic intervention descriptions and explore the suitability of the identified themes for clinical application. Clients' experiences could offer essential insights on how DMT interventions could address PD pathology and specific PD categories.

## Introduction

### Personality Disorder

People with a PD suffer from enduring inflexible patterns in cognitions and emotions, which leads to significant subjective distress affecting the self, specifically concerning identity, self-direction, and interpersonal functioning such as empathy and intimacy (American Psychiatric Association, 2013). International studies indicate that between 4.4 and 13.5% of the general adult population has at least one PD (Paris, 2010; Samuels, 2011; Quirk et al., 2016; Evans et al., 2017). Individuals with a PD are more likely to experience adverse life events, relationship difficulties, and unemployment (Tyrer et al., 2015). PDs are also associated with a severe impairment in the quality of life (Soeteman, 2008). A PD reduces life expectancy partly due to the increased risk of suicidal behaviors (Fok et al., 2012) and, one-tenth of clients with a borderline PD commit suicide (Björkenstam et al., 2016).

A PD is associated with physical health problems such as cardiovascular disease, type 2 diabetes, atherosclerosis, and hypertension, which are often under-assessed (Sanatinia et al., 2015; Tyrer et al., 2015; Evans et al., 2017). Certain personality traits are diagnosed as a PD if they are inflexible, maladaptive, persisting, and cause significant functional impairment. According to the Diagnostic and Statistical Manual of Mental Disorders (5th ed.; DSM−5; American Psychiatric Association, 2013), there are ten specific categorical types of PD divided into three clusters; Cluster A: paranoid, schizoid, schizotypal; Cluster B: antisocial, borderline, histrionic, narcissistic; Cluster C: avoidant, dependent and obsessive-compulsive. Other types are “personality change due to another medical condition” and “not otherwise specified” PD (PDNOS). The most frequent PD diagnosis is PDNOS, assigned if the general diagnostic criteria for a PD are met, but not the full criteria for any single PD (Verheul et al., 2007; Sharp and Tackett, 2014).

General criteria concern impairments in cognition, affect interpersonal relationships, and impulsivity without specifying details of impairment, while all types of PDs have an onset in adolescence or early adulthood and are stable and pervasive over time (DSM-5, American Psychiatric Association, 2013). PDs occur more often in certain populations; for instance, 60% of psychiatric clients have one or more PDs, which equals percentages found among forensic populations (Eurelings-Bontekoe et al., 2017; Evans et al., 2017). Several studies indicate that in more than 80% of the clients diagnosed with a PD, there is a co-occurrence with at least one other mental disorder, for example, a psychotic disorder, anxiety disorder, substance use disorder, eating disorder, or somatic system disorder (Cassin and von Ranson, 2005; Garcia-Campayo et al., 2007; Eurelings-Bontekoe et al., 2017). Treatment for clients consists of a range of options from cognitive behavioral therapy and psychodynamic psychotherapy to pharmacotherapy in different settings, offered over variable lengths of time (Eurelings-Bontekoe et al., 2017). DMT, one of the modalities within the Arts Therapies, is considered an established treatment in multidisciplinary mental health care for clients with a PD (Karkou and Sanderson, 2006; Dutch Mental Health Standards/Arts Therapies, GGZ Standaarden/Vaktherapeutische beroepen, 2017).

### Dance Movement Therapy and Personality Disorder

DMT is defined as the psychotherapeutic use of movement to promote emotional, social, spiritual, cognitive, and physical integration of the individual for the purpose of improving health and well-being (American Dance Therapy Association, 2018; European Association for Dance Movement Therapy, 2018). In addition, Payne et al. (2016) outline that in DMT, the emphasis is on improvised, imaginative, creative, aesthetic, and interpersonal engagement in movement. Clients with a PD are regularly referred to Arts Therapies however, the specific working mechanisms of the different arts modalities that support clients with a PD are still widely unexplored (Havsteen-Franklin et al., 2019). This becomes apparent from two recent meta-analyses that included controlled intervention studies and primary studies on DMT for a wide range of clinical populations and psychological variables (Koch et al., 2014, 2019a), with no studies specifically on DMT and PD.

A narrative literature review commissioned by the Dutch Federation of Arts Therapies (Federatie voor Vaktherapeutische Beroepen, 2017) concluded that DMT could reduce symptoms related to depression, anxiety, and stress in clients with a PD (Bräuninger, 2012; Mala et al., 2012; Koch et al., 2014). DMT could also help build a therapeutic alliance and play a role in emotion regulation and experiencing new interactions for these clients (Kil, 2010; Manford, 2014; Punkanen et al., 2014). Body awareness therapy for clients with a PD, focusing on stress management and interpersonal stress, was more effective compared to treatment as usual methods, resulting in improved body awareness and attitude toward the body as well as better self-efficacy, sleep, and physical coping resources (Gyllensten et al., 2003; Leirvåg et al., 2010). However, this narrative literature review did not deliver specific information on the applied DMT activities and the relation with the intended objectives and outcomes. The Federatie voor Vaktherapeutische Beroepen (2017) recommended conducting a systematic literature review on applied DMT interventions for clients with a PD. Consequently, the aim of this study was to conduct a systematic literature review on DMT and PD for identifying treatment themes in DMT for PD and the described DMT interventions and its components as (a) the intended therapeutic goals, (b) the therapeutic activities leading to these goals, and (c) the suggested therapeutic effects following from the activities.

## Methods

A systematic search was conducted across diverse databases following the Preferred Reporting Items for Systematic reviews and Meta-Analyses, The PRISMA Statement (Moher et al., 2009). A thematic synthesis approach was used (Thomas and Harden, 2008) to examine the content of DMT interventions for clients with a PD in detail within the retrieved articles.

### Search Strategy

The following four online databases were searched: EMBASE (MEDLINE, PubMed), WEB OF SCIENCE, PsycINFO/OVID, and SCOPUS from the inception of the databases to June 29, 2020. Reference lists of included papers were also screened to extract relevant articles. The following keywords were inserted to screen titles and abstracts: “personality disorder” AND “dance therapy” OR “dance movement therapy” OR “dance” OR “movement” OR “arts therapy” OR “arts therapies” OR “creative arts therapy.”

### Data Extraction and Eligibility Criteria

All retrieved articles were imported into RefWorks citation manager (ProQuest, 2.1.0.1), and duplicates were removed. Based on the titles and abstracts, two reviewers (1, 4) selected the articles for full-text appraisal. These reviewers independently selected articles for final inclusion based on the in- and exclusion criteria listed in Table 1. The inter-rater reliability on the decision-making on the in- and exclusion of articles was derived through Kappa statistics and was defined as: observed agreement - expected agreement/(1-expected agreement) (Orwin, 1994). When two measurements agree only at the chance level, the value of kappa is zero. When the two measurements agree perfectly, the value of kappa is 1.0. Kappa coefficients between 0.40 and 0.59 represent fair agreement, values between 0.60 and 0.74 good agreement, and values > 0.75 represent excellent agreement (Higgins et al., 2020). For an overview of the general characteristics, the following data were extracted from each study: study design and methods, setting, participants, type and frequency of the interventions, information about those leading the interventions, and measurements or assessment tools.

**Table 1:** Eligibility criteria.

	**Inclusion criteria**	**Exclusion criteria**
Study types and design	Qualitative studies, mixed-methods studies, and quantitative studies were included. There were no restrictions in study designs.	Abstracts presented in conferences, book reviews, dissertations, and brief reports
Dance movement therapy	Articles in which Dance Movement Therapy was a topic of interest and the main intervention	If DMT was considered equal to Psychomotor Therapy or other Body-oriented Therapies or articles in which dance was referred to “as having a therapeutic effect”
Publication format	Peer-reviewed published articles in English, German and Dutch	
Participants	Personality Disorder according to DSM-5 from any age, ethnicity, or gender: Cluster A: paranoid, schizoid, schizotypal; Cluster B: antisocial, borderline, histrionic, narcissistic; Cluster C: avoidant, dependent, and obsessive-compulsive	
Setting and duration	No restrictions on the setting of the interventions offered and no limitation on the length or frequency of the interventions for individuals or groups	

For extracting detailed information regarding the DMT intervention, an a priori template of codes approach was applied as outlined by Crabtree and Miller (1992). The development of the code template was informed by the Dutch Trimbos Institute (1996) and the Dutch Committee for Intervention Development Commissie Product en Module Ontwikkeling (2006), who promote the dissemination of good practices in mental health resources. They recommend that intervention descriptions for mental health care should be goal-oriented and follow a methodological and systematic therapeutic approach, with a coherent combination of problem-driven treatment and theoretical arguments based on suggested effectiveness. Fraser and Galinsky (2010) and Bartholomew Eldredge et al. (2016) suggest determining the extent to which an intervention is defined through knowledge about similar interventions and methods that have been shown to produce a significant change in similar situations. This includes explicit practice principles, activities, or objectives for changes in behavior. Following the aforementioned recommendations and encouraged by studies in the Arts Therapies on therapeutic activities and perceived effects (e.g., Haeyen et al., 2015; Odell-Miller, 2016; Havsteen-Franklin et al., 2019), resulted in the following code template consisting of three intervention components: (a) the intended therapeutic goals or change objectives, (b) the therapeutic activities leading to these goals and (c) the suggested therapeutic effects following from these activities and based on knowledge about similar interventions and methods.

### Quality Assessments

The Critical Appraisal Skills Programme (Critical Appraisal Skills Programme, 2018) for qualitative studies was used to gain insight into the quality of the articles by systematically assessing the trustworthiness, relevance, and results of the published articles. The CASP provides ten ranking items and, if all are met regarding study design and methodology, the article is considered a high-quality study. The ranking was conducted independently by two authors (1, 2). The Oxford Centre for Evidence-based-Medicine-Level of Evidence (2009) standards were used to determine the hierarchical level of best evidence by author 1. There are five different levels, with “level 1” as the highest quality of evidence consisting of case series, individual RCT's and systematic reviews of RCT's, “level 2” consists of outcome research, individual cohort studies, and systematic reviews of individual cohort studies, “level 3” are individual case-control studies and systematic reviews of case-control studies, “level 4” are case-series, poor quality cohort and case-control studies, and “level 5” consists of expert opinions without explicit critical appraisal.

### Data Analysis and Synthesis

The data analysis and synthesis were based on the thematic synthesis approach from Thomas and Harden (2008). In this first stage of thematic analysis, the selected articles were reviewed several times. To identify the intervention components concerning goals, activities, and suggested effects, line-by-line deductive coding was used for specific quotations and context to sort them in an a priori template of codes as presented by (Crabtree and Miller, 1992). This procedure was conducted independently by two authors (1, 4), and the findings were discussed until a consensus was reached. For analyzing and organizing the qualitative data, the articles were uploaded into ATLAS. ti 8 (version 8.4.4 Mac) and the quotations derived from the first stage of the thematic analysis were inserted in concordance with the a priori template of codes, the intervention components. The second stage of the thematic synthesis concerned the organization of codes without a hierarchical structure following the data-driven inductive approach of Boyatzis (1998). Quotations similar to each other in meaning and concepts were identified across all intervention components, from which interpretative descriptive themes were developed. The last stage led to the final development of overarching analytical themes representing a stage of interpretation for generating new interpretive constructs. The second and last stages were examined by author 2 for the consistency of interpretation and saturation of coding levels and in consensus with author 1 adapted.

## Results

### Article Selection

**Figure 1:**
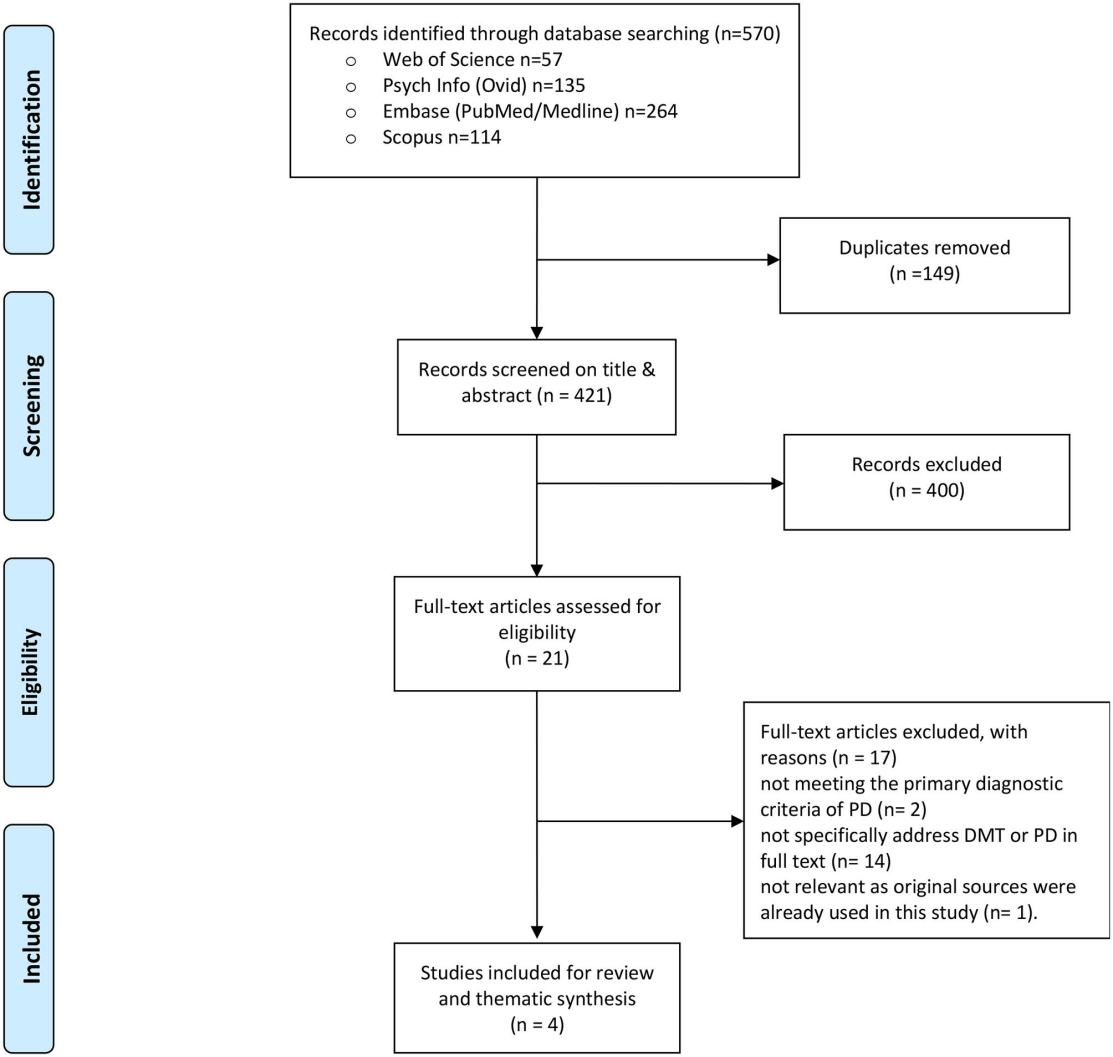
PRISMA flow diagram: article selection. From Moher et al. (2009). For more information, visit www.prisma-statement.org.

The systematic search strategy, outlined in Figure 1, led to 570 articles. After removing 149 duplicates, 421 articles were screened by title and abstract, from which 400 articles were excluded based on the eligibility criteria (see Table 1). The remaining 21 articles were retrieved for full-text appraisal. Finally, 4 articles on DMT and PD were selected. Agreement between the two reviewers regarding the inclusion and exclusion of articles was good with a Kappa value of 0.60, indicating that the observed agreement was 60% of the way between chance agreement and perfect agreement.

Seventeen articles were excluded with the following reasons: two articles (Kluft et al., 1986; Baum, 1991) addressed multiple PD which does not meet the primary diagnostic criteria of PD anymore in the DSM-5 (American Psychiatric Association, 2013), fourteen articles (Ammon, 2003; Netz and Lidor, 2003; Karterud and Urnes, 2004; Mörtl and Von Wietersheim, 2008; Cruz, 2009; Van den Broek et al., 2011; Potik and Schreiber, 2013; Jorba-Galdos, 2014; Benjamin, 2015; Pylvänäinen et al., 2015; Cobbett, 2016; Bellemans et al., 2018; Samuelsson and Rosberg, 2018; Koch et al., 2019b) did not specifically address PD and/or DMT as the main intervention. One article was excluded (Havsteen-Franklin et al., 2019) as it appeared that the study used sources (Manford, 2014; Röhricht, 2015) that were already included in the results from this study.

### General Characteristics

The included articles were published between 2013 and 2015 and were classified as a level 5 of “expert opinion” and low-quality evidence according to the hierarchy of best evidence (Oxford Centre for Evidence-based-Medicine-Level of Evidence, 2009). According to the Critical Appraisal Skills Programme (2018), no specific methodology and study design could be identified. However, the CASP analysis showed that all articles contained useful information on aims and clinical findings relevant for answering the research question concerning the content of described interventions. Details of the general characteristics are listed in Table 2. Three articles were conducted in the UK (Batcup, 2013; Manford, 2014; Röhricht, 2015) and one in the US (Pierce, 2014). The case report from Manford (2014) addressed one client with only a borderline PD. The three other articles addressed borderline PD with at least one additional PD category, accompanying symptoms or comorbidity with another mental disorder. Pierce (2014) focuses on clients with dissociative symptoms also seen in clients with a PD. Röhricht (2015) discusses severe mental illnesses, including PD. Two studies concerned clients with a PD in a forensic setting (Batcup, 2013; Manford, 2014). The articles from Manford (2014) and Pierce (2014) offered most information on how dance movement therapists work with clients with a PD. Manford (2014) describes in detail how certain activities in DMT were executed. Pierce (2014) offers a 3-phased model for DMT with interventions for safety and stability, the integration of traumatic memories, and the development of the relational self and rehabilitation. Both these articles were related to attachment theory and trauma psychology. Röhricht (2015) and Batcup (2013) offered a broad overview of different populations in mental health care settings, including clients with a PD. Manford (2014), the only DMT interventionist identified, holds a master's degree in DMT. No assessment tools for measuring outcomes could be identified.

**Table 2:** General characteristics of included articles.

**Author Year Country**	**Research types**	**Method/design and aim of study**	**Setting**	**Diagnosis according to DSM-5 (American Psychiatric Association, 2013)**	**CASP rating**	**Levels of evidence**
Batcup (2013) UK	Qualitative/ Expert opinion	Theoretical discussion of Dance Movement Psychotherapy (DMP) literature relative to psychiatric diagnosis, trauma, violence, abuse, and growing evidence in the form of RCTs, empirical research, government guidelines, surveys, audits, case studies, and unpublished data	Forensic settings	Borderline PD Antisocial PD	3	5
Pierce (2014) USA	Qualitative/ Expert opinion	Theoretical discussion of the contributions of trauma psychology and DMT on dissociation; and an attachment-oriented framework is offered for developing interventions to support DMT-therapists in using the integrative power of DMT with dissociation (& PD)	N/A	Borderline PD with symptoms of dissociation of somatic, emotional, and psychological experiences related to traumatic events	3	5
Manford (2014) UK	Qualitative/ Case report	Case study: time-limited DMP with a female offender diagnosed with borderline PD looking particularly at the development of the therapeutic relationship and attachment theory	Secure hospital environment	One client diagnosed with borderline PD with an offense for fire setting at home	3	5
Röhricht (2015) UK	Qualitative/ Expert opinion	Theoretical overview of how Body Psychotherapy, including DMP, is utilized for the treatment of a range of severe mental disorders.	N/A	Borderline PD; Eating disorder with a borderline organization; Borderline PD in co-occurrence with schizophrenia, Narcissistic PD, Schizoid PD	3	5

### Data Analysis of Intervention Components Per Article

One hundred fifty-six quotations were identified related to the content of DMT interventions, the components: goals, therapeutic activities, and suggested effects. Table 3 shows that all intervention components were identified in all articles and that the ratio of quotations related to the components differed considerably in every article and across all articles. In the articles, the three intervention components were described rather implicitly, and no methodological line, a full series of components could be identified, which was finally distilled through thematic analysis and synthesis. Each article also had a different focus, which influenced the quantifications of the intervention components per article. In the article from Batcup (2013), most quotations concerned suggested effects. This is in line with the broad overview of the presented evidence of a wide range of research data on different clinical populations and symptoms. In Pierce's (2014) article, most of the quotations concerned goals and activities, which paired with the presented theoretical discussion for creating a base for a phase-oriented framework with DMT activities. In the article from Röhricht (2015), the largest number of identified quotations was related to goals for clients with a severe mental illness, including a PD. Röhricht (2015) argues that there was only a very limited body of literature and no evidence from clinical trials on PD, which may have affected the low number of identified quotations related to activities and effects in this area. The most balanced ratio of quotations related to all intervention components was identified in Manford case report 2014.

**Table 3:** Overview of intervention components per article.

**Intervention components**	**Pierce (2014)**	**Röhricht (2015)**	**Manford (2014)**	**Batcup (2013)**
Goals 52	26	11	12	3
Activities 52	27	4	12	4
Effects 49	16	2	13	18
Total				
148 Quotations	70	17	37	26

### Thematic Synthesis From Descriptive to Analytical Themes

From the thematic synthesis, 15 overarching descriptive themes were constructed, resulting in the following six overarching analytical themes: Self-regulation (57 quotations); Interpersonal relationships (36 quotations); Integration of self (25); Processing experiences (15); Cognition (13) and Expression and Symbolization in Movement and Dance (10) (see Table 4). All three intervention components, goals, therapeutic activities, and suggested effects were identified within the analytical themes of self-regulation, interpersonal relationships, and cognition, whereas this was not the case for the other analytical themes. No activities could be identified for the analytical themes, integration of self, and processing experiences, while no goals were identified for expression and symbolization in movement and dance.

**Table 4:** Overview of descriptive and analytical themes.

**Descriptive themes**			**Analytical themes**
**Goals**	**Activities**	**Suggested effects**	
Regulation of emotions and thoughts (16)	Awareness and regulation (22)	Regulation of emotions and thoughts (10)	Self-regulation (49)
Relational engagement (12)	Shared movement (10)	Relational engagement (14)	Interpersonal relationships (36)
Integration of self (13)	———	Integration of self (12)	Integration of self (25)
Non-verbally processing experiences including (developmental) trauma (5)	———	Non-verbally processing experiences including (developmental) trauma (10)	Processing experiences (15)
Mentalization/Mindfulness and thinking (4)	Thinking and verbalization (7)	Thinking and verbalization (2)	Cognition (13)
——–	Expression and symbolization in movement and dance (9)	Expression and symbolization in movement and dance (1)	Expression and symbolization in movement and dance (10)
Total			
50	48	50	148 Quotations

#### Self-Regulation

Most quotations concerned the client's awareness and the regulation of emotions and thoughts, leading toward the overarching analytical theme of self-regulation. Self-regulation, a major goal emphasized by all authors, supports clients in tolerating internal experiences, including emotions and thoughts while moving. Within this context, Pierce (2014) refers substantially to the field of trauma psychology (e.g., Ogden et al., 2006a; Schore, 2009; Porges, 2011; Siegel, 2012). Pierce (2014) claims that attention to interoceptive awareness is of major importance by developing clients' skills for orienting themselves in present time and space supported by identifying different somatic markers during DMT. In the first phase of treatment, a client creates a personal arousal scale and action plan, including relaxation exercises and working with touch for down and up-regulation during hyper- and hypoarousal (Pierce, 2014). In addition, psychoeducation and developing the capacity to recall and think about movement is essential in regulating and controlling tension, impulses, and aggressive behaviors (Batcup, 2013; Manford, 2014; Röhricht, 2015). Body-oriented activities are commonly offered at the beginning of treatment for regulating emotions and thoughts, such as grounding exercises and attention to 5-sense perception and breathing patterns (Manford, 2014; Pierce, 2014; Röhricht, 2015). For self-regulation and maintenance of movement and connectedness, the use of props is proposed to support gross sensation and motor movement (Manford, 2014; Pierce, 2014). Techniques were described as mirroring and self-referential movements, such as pressing the feet into the floor for clients with dissociative symptoms (Pierce, 2014). Batcup (2013) suggests that through creating a dance, embodiment, and verbal reflection, the ability to mentalize and remain mindful and focused in the present is expanded. Pierce (2014) and Röhricht (2015) mention the use of Laban Movement Analysis and the Kestenberg Movement Profile as systematic formats for observation of movement patterns and for expanding the movement repertoire to support self-regulation. Pierce (2014) suggests specific DMT approaches as Authentic Movement (Adler, 1999; Avstreih, 2008) and Caldwell's Moving Cycle 1996 to support clients' ability to non-judgmentally track inner experiences across somatic, cognitive, and emotional domains. Through the therapeutic relationship and guided movement experiences, clients could be supported in expressing and structuring their thoughts and feelings in a regulated way assisted by cognitive, verbal reflection (Pierce, 2014). A suggested effect is that DMT could offer access to mindfulness through the non-judgmental observation of sensation, emotion, and movement while increasing the “capacity to think” (Manford, 2014; Pierce, 2014). DMT could also support the exploration of alternative approaches for managing emotions less destructively (Manford, 2014; Röhricht, 2015).

#### Interpersonal Relationships

In all of the articles, the conclusion was drawn that the client must feel safe with others in DMT for achieving successful therapy outcomes. The often disturbed early dyadic attachments, which affected the client's capacity to relate to others in a healthy way, can be repaired through the interactive regulation between the dance movement therapist and the client as in early parent-child non-verbal communication. Pierce (2014) suggests that this process could be supported by a dance movement therapist with a broad movement vocabulary and strong observational skills for repatterning the quality of the presented affect in the client. Pierce (2014) further suggests that the development of relational skills and authentic relational engagement is supported by focusing on shared movement experiences among group members in dance, rhythm, and creative and spontaneous movement interactions. It creates empathy and promotes openness to each other's experiences while overcoming resistance (Batcup, 2013; Pierce, 2014; Röhricht, 2015).

Many activities for relational engagement were identified. Pierce (2014) offers a large toolbox with activities and techniques such as mirroring, interactive experiential movement, and exploring this through imitation, synchronous movement, and motoric cooperation. Manford (2014) describes how props are used to create interpersonal connections. Manford (2014) and Pierce (2014) suggest that DMT may effectively treat dysfunctional relating patterns through mirroring, attunement, and simple shared creative and spontaneous movement and dance experiences. According to Batcup (2013), DMT facilitates engagement and builds an alliance in shared language while contributing to more social behaviors and bonding. Batcup (2013) and Manford (2014) suggest that in DMT, clients can strengthen their capacity to think about themselves and others while exploring alternative behaviors.

#### Integration of Self

All articles relate the origin of PD to early relational-attachment trauma, which can cause dissociation, projective identification, as well as unconscious, splitting off from painful experiences, resulting in a declined capacity of clients with a PD to integrate frustrating but also satisfying experiences (Manford, 2014; Röhricht, 2015). According to Pierce (2014), a goal in DMT is to support the integration of memories and self-states that clients previously could not tolerate, especially when it concerns clients with trauma-related dissociative symptoms. Supported by concepts from affective neuroscience (e.g., Cozolino, 2002; Ogden et al., 2006b; Schore, 2009; Siegel, 2009), Pierce (2014) introduces the role of right-brain dominance during traumatic memory recall and views dissociation as a disintegration of right brain regulatory functions serving as a defense against internal sensations too painful to tolerate. This results in a less coherent sense of self or a pathological, dissociated, and defended self (Batcup, 2013; Pierce, 2014). Pierce (2014) suggests that processing metaphors and symbols recruit the right brain at a preconscious and often nonverbal level, stimulating connections between the integrative neural networks, which provides the foundation for a secure and cohesive sense of self. Röhricht (2015) claims that as clients with a PD have an unclear sense of body-ego boundaries and a lack of coherent self-identity, there is not enough ego control to integrate experiences. A goal is to encourage ego-maturation processes retrospectively with non-verbal communication, interaction, and empathy while directly addressing the clients' split-off experiences (Röhricht, 2015). Furthermore, clients are encouraged to move authentically while tracking and processing emotional, cognitive, and somatic experiences that occur within the body (Manford, 2014; Pierce, 2014), which contributes to positive change and the reconstruction of a more integrated and coherent sense of self (Batcup, 2013; Manford, 2014). A suggested effect mentioned by Pierce (2014) is that DMT may provide direct access to bottom-up integration of the right brain, which supports the processing of implicit information.

#### Processing Experiences

An important goal emphasized in all of the articles is to support clients in processing past and present experiences that surface in DMT, thus facilitating the re-establishment of trust, intimacy, social skills, and self-esteem. According to Pierce (2014) and Röhricht (2015), addressing such experiences and clients' specific biographic context by creating coherent narratives and making sense of them can only take place once clients have established a certain degree of self-regulation. Only then can subsequent resourcing, sequencing, and integration of past traumatic experiences on a bodily level take place (Pierce, 2014; Röhricht, 2015). According to Pierce (2014), a suggested effect is that DMT supports the processing of metaphors and symbols that recruit the right brain at a preconscious level, assisting the integration and resolution of psychological trauma. Here Pierce (2014) refers to Johnson (1987), who suggests “that symbolic imagery, sound, and gesture provide transitional spaces” (p. 11) that afford safe distancing and a symbolic realm to engage in traumatic material. This would support clients in thinking about and understanding their past experiences. According to Batcup (2013), DMT offers the necessary distancing and the simultaneous containment of disclosing traumatic events and may also enable the discovery of very early embodied knowledge not available before. DMT also offers an approach to psychotherapy that enables people with learning difficulties or disabilities to gain easier access through non-verbal communication or a shared language (Batcup, 2013).

#### Cognition

Batcup (2013) and Pierce (2014) suggest including mentalization, verbal reflection and, validation in DMT to increase the client's capacity to think about their own and others' mental states in present moments. Mentalizing capabilities are understood to be a result of secure attachment, a base for developing self-regulation skills for emotional and somatic arousal (Pierce, 2014). In this process, mindful self-awareness is an important goal for relating to experiences as purposeful autonomous means instead of holding on to habitual beliefs and behaviors (Batcup, 2013; Pierce, 2014). According to Manford (2014), verbal reflections support the maintenance of these experiences, and words can accompany the clients' movements to recall, think about and make sense of them. The dance therapist's verbal validation, including direct verbal addressing of split-off experiences of the client, offers support in reducing impulse control (Batcup, 2013; Manford, 2014; Röhricht, 2015).

#### Expression and Symbolization in Movement and Dance

According to Batcup (2013) and Pierce (2014), improvisational and free-associative movement interventions support the access and expression of preconscious material directly through the body, while symbolization and the (re-)structuring of narrative material supports the thinking about and the understanding of such an experience. Manford (2014) offers many examples of this approach while making use of metaphors and props. Pierce (2014) highlights specific DMT approaches as Halprin's “expressive arts chorus” 1999 and Gray's “body stories” 2001 to access and express preconscious material. With reference to (Berger, 2012, as cited in Pierce, 2014, p. 11), it is suggested that dance allows individuals to re-create themselves through the use of symbolism and expression. Also, creative expression in dance could support clients in expanding their expressive movement repertoire, which was stalled due to suppression (Pierce, 2014). Batcup (2013) refers to kinaesthetic and kinetic imagery (Dosamantes-Alperson, 1983), which could be considered a way to achieve a compassionate, artistic distance from the personal experience of disturbing life stories. Both Batcup (2013) and Pierce (2014) state that the use of dance, symbolism, and expression may eventually create a more bearable organization of thoughts and associated emotions.

## Discussion

### General Characteristics of the Articles

This study brings together available information on DMT for clients with a PD. The four reviewed articles are all expert opinions and offer essential information on specific themes to focus upon in DMT when working with clients with a PD. Nevertheless, more and higher-quality studies are necessary to explore if DMT is an effective intervention for these clients and which factors contribute to positive change. A few general characteristics of the articles are noteworthy. The main focus in all of the articles was on borderline PD, which is consistent with the fact that most studies on PD concern a borderline PD (Eurelings-Bontekoe et al., 2017; Hutsebaut et al., 2018). In agreement with conclusions from experts in the field of PD (Cassin and von Ranson, 2005; Garcia-Campayo et al., 2007; Eurelings-Bontekoe et al., 2017), at least one additional PD category, accompanying symptoms or comorbidity with another mental disorder, was described. Comparable with the high percentages of PD's found among forensic populations (Eurelings-Bontekoe et al., 2017; Evans et al., 2017), information on this client population was well-represented (Batcup, 2013; Manford, 2014).

### The Themes and Their Implications for DMT Interventions for Clients With a PD

Although the reviewed articles did not systematically describe a DMT intervention, specific information on the applied DMT activities in relation to the intended goals and suggested effects could be composed from thematic synthesis. A full series of intervention components were identified across the articles for the analytical themes, self-regulation, interpersonal relationships, and cognition. These synthesized methodical lines offer consistent, essential, and detailed information, which is mandatory for systematically describing interventions for treatment guidelines and research (Fraser and Galinsky, 2010; Bartholomew Eldredge et al., 2016). The themes of self-regulation, interpersonal relationships, and cognition also correspond with the general criteria of PD concerning impairments in cognition, affect, interpersonal relationships, and impulsivity (American Psychiatric Association, 2013). Compared to the themes of self-regulation and interpersonal relationships, only a small number of quotations were related to the theme of cognition, which encompasses mentalization, mindful attention, and recalling and thinking about movement. These approaches seem in service of self-regulation and the understanding of own and other people's experiences. All these cognition-based approaches are firmly rooted in psychological theoretical constructs, while interventions informed by the arts, including dance (Samaritter, 2018), were not explicitly described as possible ways for reinforcing self-regulation. It was recommended to first focus on body-oriented activities and cognitive strategies for self-regulation before touching on more dynamic dance-informed structures (Manford, 2014; Pierce, 2014; Röhricht, 2015). A reason for this, referred to in all of the articles, is the concern that clients with a PD do not possess enough impulse control yet at the beginning of the treatment to integrate experiences derived from such approaches. This reasoning is in line with the results, showing that dance-informed interventions as expressing biographic material, symbolic movement, or creation were mainly applied for processing experiences and self-integration. Valuable for using dance-informed interventions for self-regulation in DMT are the considerations from Batcup (2013), Manford (2014), and Pierce (2014). They describe how DMT offers a transitional space and a symbolic realm for clients to fully engage in while at the same time an artistic distance is created from painful experiences. Following this line of thought, a hypothesis could be that clients' regulation of emotions and thoughts also takes place once clients are engaged in art/dance. A concept that was barely elaborated on and further exploration of how dance-informed interventions can support the clients' self-regulative functioning with a PD is suggested.

### Strengths and Limitations

This is the first systematic review of published articles on DMT and PD, which includes detailed information for promoting DMT as a possible effective treatment for clients with a PD. The chosen methodology focusing on identifying intervention components appeared to be very useful to systematically analyze, identify, quantify and synthesize qualitative content. It also revealed important change objectives to focus on in DMT for PD and offered substantial and in-depth information regarding DMT interventions for PD. The retrieved data from this study can support the development of systematically describing interventions essential for the purpose of treatment guidelines and research. Certain findings from studies on body-oriented approaches that were excluded for review might have supported the effectiveness of DMT for clients with a PD. Nevertheless, the choice was made to focus on DMT only to clarify what is available within this modality. Consequently, this approach revealed the limited information available, with the absence of strong evidence for prescribing DMT for clients with a PD. This shortage of findings on DMT and PD limits the findings' accountability. Notably, the focus in the expert studies is mainly on clients with a borderline PD or comorbid with another PD, and therefore the results cannot be generalized for all PD categories.

### Recommendations

The intervention content consisting of “goals-activities –effects” was rather implicit in the reviewed articles. Systematic descriptions of DMT interventions for clients with a PD would be needed to support the application, evaluation, and replicability for clinical practice and research. Future research on practitioners' expertise of the application of DMT interventions for PD could support the development of systematic intervention descriptions, while the suitability of the identified themes for the clinical application of DMT with PD could be explored. The clients' experiences of the DMT could offer essential insights on how DMT interventions should be tailored to address PD pathology. Clinical studies may provide more insight into the applicability of specific DMT interventions including dance-informed interventions, and the effective working factors for PD and specific PD categories.

## Conclusions

This systematic review and thematic synthesis of expert articles on DMT and PD offers substantial information on treatment themes to focus upon as also the intended therapeutic goals, therapeutic activities leading to these goals, and the suggested therapeutic effects following from these activities in DMT for PD. A full series of intervention components, goals-activities-effects, offers fundamental ingredients for developing systematic intervention descriptions with a consistent methodological line for treatment guidelines and research. Such consistency was identified in the analytical themes of self-regulation, interpersonal relationships, and cognition, which are suggested major themes to focus upon in DMT for PD. These themes are closely related to the general criteria of PD pathology concerning the areas of cognition, affect, interpersonal relationships, and impulsivity. The findings also show that dance movement therapists apply an integrative approach in the treatment of PD, with alternations of body-oriented and dance-informed activities. Notably, the findings showed that the use of body-oriented approaches and cognitive strategies was in favor of dance-informed approaches for self-regulation.

## Author Contributions

SK developed the research design, conducted the research, the thematic analysis, and synthesis, and conceptualized it. RS and JS supervised the review process and co-authored the manuscript. SK and GK conducted the data extraction independently. RR supported the search strategy. RS and SK independently assessed the quality of the articles and cross-validated the thematic synthesis. All authors have contributed toward the manuscripts' revisions and read and approved the submitted version.

## Conflict of Interest

The authors declare that the research was conducted in the absence of any commercial or financial relationships that could be construed as a potential conflict of interest.
